# Genome-Wide Identification of PEBP Gene Family in Two *Dendrobium* Species and Expression Patterns in *Dendrobium chrysotoxum*

**DOI:** 10.3390/ijms242417463

**Published:** 2023-12-14

**Authors:** Meng-Meng Zhang, Xuewei Zhao, Xin He, Qinyao Zheng, Ye Huang, Yuanyuan Li, Shijie Ke, Zhong-Jian Liu, Siren Lan

**Affiliations:** 1 College of Forestry, Fujian Agriculture and Forestry University, Fuzhou 350002, China; 1220428020@fafu.edu.cn (M.-M.Z.); zxw6681@163.com (X.Z.); 5220422102@fafu.edu.cn (X.H.); 3210422027@fafu.edu.cn (S.K.); 2 Key Laboratory of National Forestry and Grassland Administration for Orchid Conservation and Utilization, College of Landscape Architecture and Art, Fujian Agriculture and Forestry University, Fuzhou 350002, China; qinyaozheng@fafu.edu.cn (Q.Z.); 5221726097@fafu.edu.cn (Y.H.); lyy9902140575@163.com (Y.L.)

**Keywords:** PEBP genes, *D. chrysotoxum*, *D. nobile*, phylogenetic analysis, expression analysis

## Abstract

The PEBP gene family plays a significant role in regulating flower development and formation. To understand its function in *Dendrobium chrysotoxum* and *D. nobile* flowering, we identified 22 PEBP genes (11 *DchPEBP*s and 11 *DnoPEBP*s) from both species. We conducted analyses on their conserved domains and motifs, phylogenetic relationships, chromosome distribution, collinear correlation, and *cis* elements. The classification results showed that the 22 *PEBP*s were mainly divided into three clades, as follows: FT, MFT, and TFL1. A sequence analysis showed that most PEBP proteins contained five conserved domains, while a gene structure analysis revealed that 77% of the total PEBP genes contained four exons and three introns. The promoter regions of the 22 *PEBP*s contained several *cis* elements related to hormone induction and light response. This suggests these *PEBP*s could play a role in regulating flower development by controlling photoperiod and hormone levels. Additionally, a collinearity analysis revealed three pairs of duplicate genes in the genomes of both *D. chrysotoxum* and *D. nobile*. Furthermore, RT-qPCR has found to influence the regulatory effect of *DchPEBP*s on the development of flower organs (sepals, petals, lip, ovary, and gynostemium) during the flowering process (bud, transparent stage, and initial bloom). The results obtained imply that *DchPEBP8* and *DchPEBP9* play a role in the initial bloom and that *DchPEBP7* may inhibit flowering processes. Moreover, *DchPEBP9* may potentially be involved in the development of reproductive functionality. *PEBP*s have regulatory functions that modulate flowering. FT initiates plant flowering by mediating photoperiod and temperature signals, while TFL1 inhibits flowering processes. These findings provide clues for future studies on flower development in *Dendrobium.*

## 1. Introduction

Phosphatidylethanolamine binding protein (PEBP) is from a highly conserved domain [1] that regulates flowering transition and plant architecture [2]. This gene can affect the regulation of photoperiod, vernalization, environmental temperature, plant hormones, and autonomous flowering pathways [3,4,5,6]. Phosphatidylethanolamine binding proteins were initially extracted from bovine brains, due to their high level of attraction towards phospholipids [7,8]. The PEBP gene family is widespread in bacteria [9], yeast [10], plants [11], and animals [1,12]. In higher plants, the PEBP gene family is categorized into three subfamilies: MOTHER OF FT AND TFL1 (MFT) like, FLOWERING LOCUS T (FT) like, and TERMINAL FLOWER 1 (TFL1) like. These subfamilies, namely MFT-like and FT-like, either promote or inhibit flowering processes [13]. There are multiple PEBP family genes in different plants, and although researchers have some understanding of their role in plant development, this understanding is limited. To better understand the mechanism of PEBP family genes in plants, further research is required to study their functions in different plant species.

PEBP proteins play a crucial role in various developmental processes in plants. In previous studies, the flowering site T (FT) [14] was found in *Arabidopsis Thaliana* to induce flowering in a photocycle-dependent manner, while their homologous terminal flower 1 (TFL1) gene family PEBP amine-binding protein (PEBP) [15] delayed flowering. Homologs of FT and TFL1 (MFT) have been identified in bryophytes lacking other FT and TFL1-like genes [16]. Mixed FT/TFL1 genes, as well as MFT-like genes, can be found in gymnosperm species [13]. It is suggested that MFT-like genes are the ancestors of FT-like genes and TFL1-like genes. Understanding of the separation function of FT and TFL1 evolved after the differentiation of gymnosperms and angiosperms [17]. The gene FT was found to be up-regulated in the reproductive organs of the *Dendrobium* ‘Chao Praya Smile’ orchid, including inflorescence apices, stems, floral buds, and open flowers. When overexpressed, the *DoFT* gene promotes flowering, resulting in notable pseudobulb formation, while suppressing the endogenous *DoFT* transcripts delays flowering in orchids [18]. The TFL-like gene is down-regulated in adult *D. catenatum*, suggesting a mechanism is present that inhibits flowering during the vegetative growth phase in rattan plants [19].

Orchidaceae is a diverse family of flowering plants with ecological and economic importance. About 27,800 species are widely distributed around the world [20]. *Dendrobium* is a highly abundant genus within the orchid family, with about 1100 species found worldwide [21]. The PEBP gene family plays important roles in plant growth; for example, flowering, organ development, seed germination, root morphology, and resistance to abiotic stress [18,22]. However, most of the PEBP gene functions in *D. chrysotoxum* and *D. nobile* remain unclear. Studying the evolutionary features and expression profiles of *PEBP*s can enhance our understanding of their functions in two *Dendrobium* species. The number of PEBP gene families in plants varies significantly. To date, six, seven, and five *PEBP*s have been identified in dicotyledonous plants such as *Arabidopsis* [23], apple [14], and grape [24], respectively, and 24 and 19 *PEBP*s have been identified in monocotyledonous plants such as maize [25] and rice [26]. In addition, *DhPEBP*s [27] in *D. huoshanense* have various regulatory functions responsible for modulating flowering. FT orthologs from *D.* ‘Chao Praya Smile’ [18] correlate with flower development and accelerate flowering.

This study aimed to investigate the characteristics of the PEBP gene family during orchid floral development by performing gene structure analysis, phylogenetic tree construction, collinearity analysis, *cis*-acting element analysis, and expression pattern analysis in *D. chrysotoxum* and *D. nobile*. Understanding the regulatory function of the PEBP gene family in the flower organ development processes of different orchid life forms is crucial for the breeding and development of orchids. The findings of this study will provide a scientific basis for understanding the distinct roles of PEBP family members in the flowering regulation of both *Dendrobium* species.

## 2. Results

### 2.1. Identification and Physicochemical Properties

Using 6 PEBP gene family protein sequences of *Arabidopsis*, 22 PEBP gene family members were identified in the *D. chrysotoxum* and *D. nobile* genome using the BLAST and HMMER techniques. The PEBP proteins are named according to their order of gene distribution on the chromosome, starting at the top and going down (Table S1). The PEBP proteins have varying physicochemical properties, ranging from 82 to 420 amino acids. Molecular weight (Mw) ranges from 9.64 to 48.32 kDa. In addition, 7 of the 22 *PEBP*s were basic proteins (isoelectric point (pI) above 8.00) and 15 were neutral or weakly acidic proteins (pI range 4.99 to 7.87). The 22 PEBP proteins were all predicted to be hydrophilic, with a negative total mean of hydrophobicity (GRAVY). A total of 22 PEBP genes were named *DchPEBP1*-*11* or *DnoPEBP1*-*11*, according to their order of distribution on the chromosome. In addition, the analysis of coding protein sequences showed that there were significant differences in amino acid, molecular weight, pI, GRAVY, AI, and II of the PEBP gene in *D. chrysotoxum* and *D. nobile.*

### 2.2. Phylogenetic and Classification of PEBP Proteins

**Figure 1:**
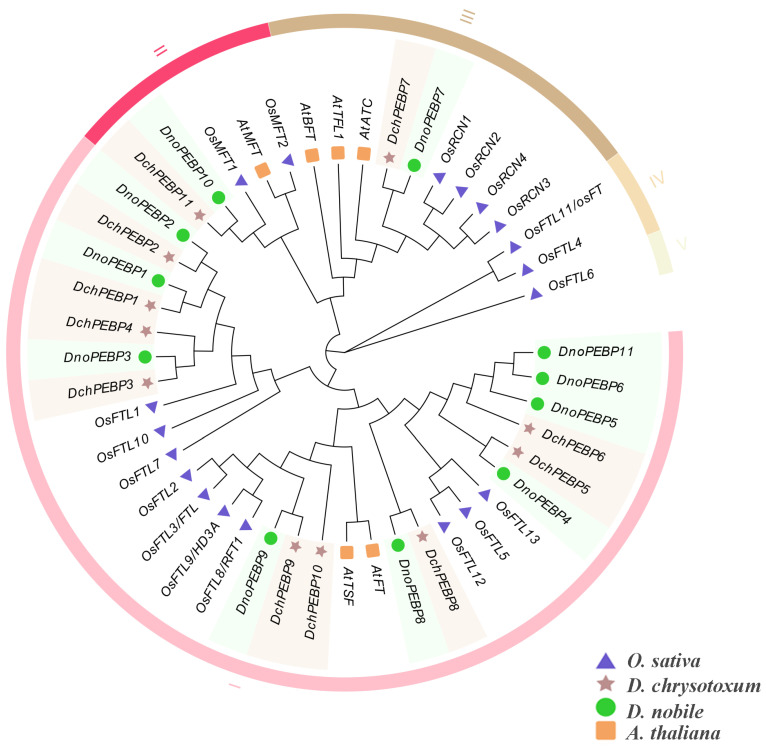
Phylogenetic tree of *PEBP*s in four plants. The PEBP gene family is divided into five subfamilies (I–V), and the IV and V subfamily does not contain the *PEBP*s of *D. chrysotoxum* and *D. nobile*. The PEBP protein sequence of *D. chrysotoxum* and *D. nobile* can be obtained in Table S1.

A phylogenetic tree was constructed to show the relationship between *PEBP*s in *D. chrysotoxum* and *D. nobile*. The tree was based on the protein sequences of 45 members of PEBP from *D. chrysotoxum* (*DchPEBP*), *D. nobile* (*DnoPEBP*), *A. thaliana* (*AthPEBP*), and *Oryza sativa* (*OsPEBP*), which were divided into 5 classes (Figure 1). *D. chrysotoxum* and *D. nobile* belong to I, II and III, IV and V are rice genes. After analyzing the classification of *AthPEBP*s, the 22 PEBP genes in the 2 *Dendrobium* species were categorized into three subfamilies. The resulting phylogenetic tree was also divided into three subfamilies: FT(I), MFT (II), and TFL1 (III, IV, V). There were 18 genes in the FT subfamily, 9 nine *DchPEBP*s and 9 *DnoPEBP*s. Additionally, there were two genes in the TFL1 subfamily (*DchPEBP7* and *DnoPEBP7*) and two genes in the MFT subfamily (*DchPEBP11* and *DnoPEBP10*).

### 2.3. Phylogenetic Analysis and Conserved Motifs of PEBP Proteins

**Figure 2:**
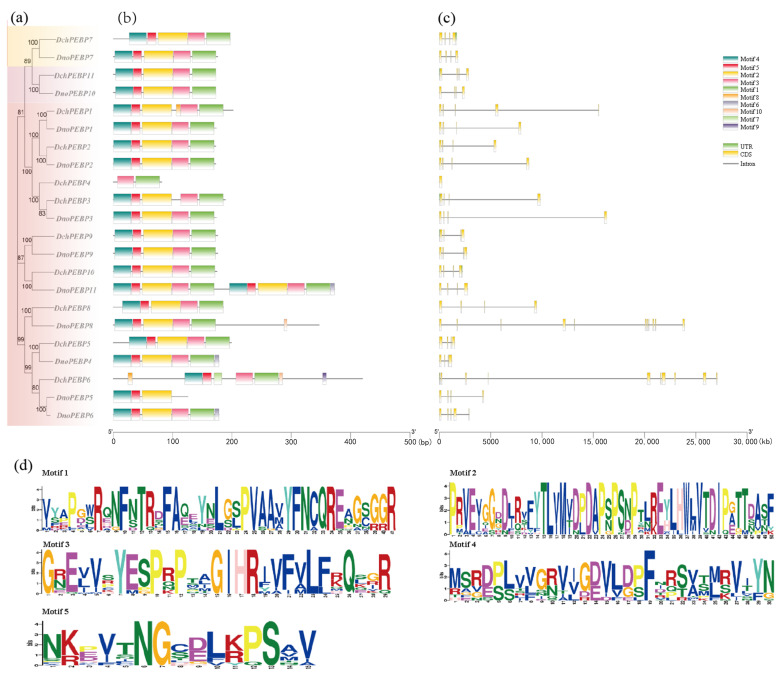
Phylogenetic relationships, motif, and structure of *PEBP*s in *D. chrysotoxum* and *D. nobile*. (**a**) MEGA7.0 was used to construct a phylogenetic tree of 22 *PEBPs*; (**b**) used the conserved motif of the predicted PEBP proteins on MEME; (**c**) gene structure of the PEBPs of *D. chrysotoxum* and *D. nobile*; (**d**) conserved domains of *D. chrysotoxum* and *D. nobile* species protein sequences.

To study the features of the PEBP proteins, we used the motif-based sequence analysis tools database to analyze the conservative domains of 22 PEBP proteins using Multiple Em for Motif Elicitation (MEME). The conservative domains were set from motif1 to motif10. The sequences of the motifs identified were then characterized (Figure 2b). The distribution of conserved domains among proteins in the same branch was found to be similar. The TFL1 subfamily contains the most complete conserved motif, followed by the MFT subfamily. Motif1 to motif5 are present in the majority of PEBP proteins, suggesting that they may have similar functions. Deletion of motifs varies among subfamilies. *DchPEBP4* has only motif1 and motif3, while *DnoPEBP5* has motif2, motif4, and motif5. This may result in the loss of some functions (Figure 2d). Moreover, most of the 22 PEBP genes contain 3 introns, although a very limited number have 9 to 10 introns (Figure 2c). The evolutionarily conserved genetic structure of the PEBP gene family showed that 80% of genes have four exons and three introns.

### 2.4. Genes Expression Analysis of the PEBP Genes

**Figure 3:**
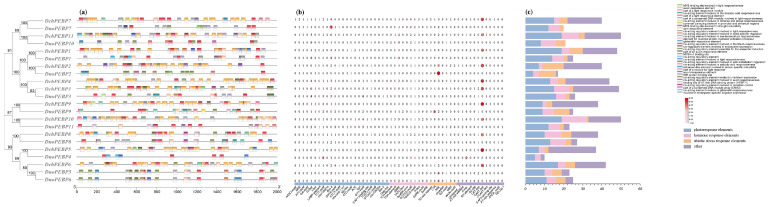
Regulatory elements in the promotor region of two *Dendrobium* species. (**a**) The *cis*-acting elements of *D. chrysotoxum*; (**b**) the number of *cis*-acting elements in the promoter region; (**c**) count the number of photoresponse elements, hormone response elements, abiotic stress response elements, and others for each PEBP gene. The captions are marked on the right, and the types and quantities of *cis*-acting elements are shown in Table S2.

We investigated the regulatory function of *PEBP*s by searching for *cis* elements in the promoter region of 22 genes from two *Dendrobium* species. Throughout the study, we obtained a total of 35 *cis* elements, for a total of 720 *cis* elements (Table S2). Ten common elements exist among most *PEBP*s, with variations providing diverse gene functions. In this study, *DchPEBP10* contained 50 *cis* elements, which is the largest number (Figure 3a). Through analysis of the promoter, it was found that there are typical light response elements AE-box, Box 4, G-box, and TCT-motif on the PEBP promoter. Light responses are photoreactive elements with the largest number (264/720), which indicates that the regulation of PEBP function by light is particularly important. In addition, among the identified elements, hormone response elements related to flowering regulation were found, such as GARE elements. The types and quantities of light response-related elements were the largest, followed by *cis* elements related to plant hormone regulation and stress-related *cis* elements (Figure 3b). It is therefore suggested that *PEBP*s may play a role in resistance to abiotic stress.

According to the characteristics of *cis*-acting elements, these elements can be divided into three categories: hormone response elements, photoresponse elements, abiotic stress response elements, and others (Figure 3c). The number of photoresponsive elements is significantly higher than that of other regulatory elements. We hypothesized that the *PEBP*s of *D. chrysotoxum* and *D. nobile* play an important role in light response regulation, so we hypothesized that they also play a role in regulating the flowering period.

### 2.5. Chromosomal Localization and Collinearity Analysis of PEBPs

**Figure 4:**
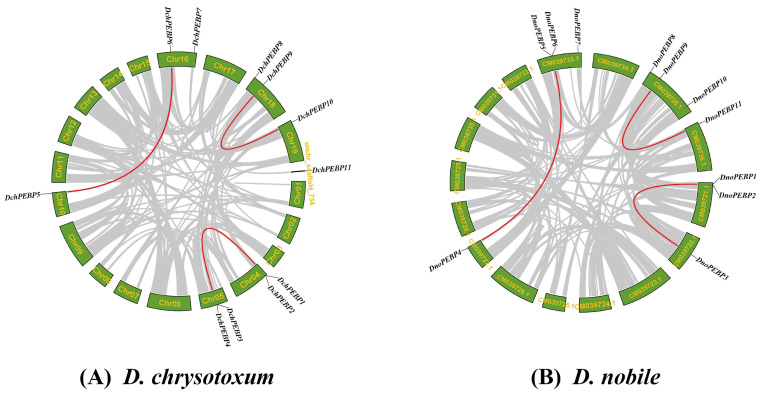
Synteny analysis of *PEBPs* in two *Dendrobium* species. (**A**) Synteny analysis of *DchPEBP*s. (**B**) Synteny analysis of *DnoPEBP*s. Red lines represent segmentally duplicated gene pairs.

TBtools was used to visualize the distribution of 22 *PEBP*s on chromosomes. The results showed that the PEBP of the two species of *Dendrobium* were distributed on different chromosomes (Figure S1). Eleven *DchPEBP*s were distributed on seven chromosomes, of which *DchPEBP11* was distributed on unknown chromosomes. Eleven *DnoPEBP*s were distributed on six chromosomes. Three pairs of fragment repeat genes were found in *D. chrysotoxum* (Figure 4A) *DchPEBP1* and *DchPEBP2*, *DchPEBP3*, and *DchPEBP4*, among which two pairs were tandem repeats. There were also three pairs of fragment repeats identified in *D. nobile* (Figure 4B), and interestingly, there were also two pairs of tandem repeats: *DnoPEBP1* and *DnoPEBP2*, *DnoPEBP5* and *DnoPEBP6*. This suggests that a gene replication event occurred during the evolutionary process.

### 2.6. Expression Patterns of PEBP Genes in D. chrysotoxum

**Figure 5:**
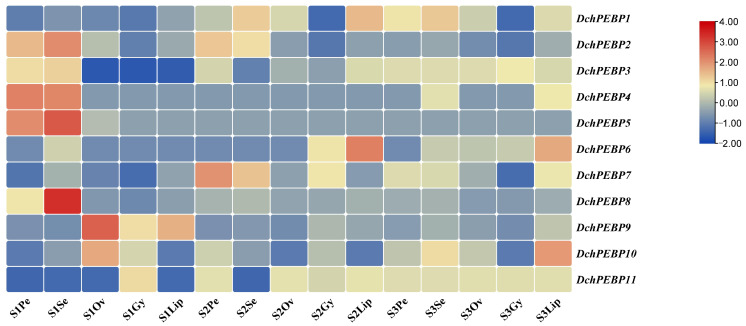
The expression pattern of *PEBP*s in the floral component of *D. chrysotoxum*. Expression pattern of *PEBP*s in flower components (Pe: petals; Se: sepals; Ov: ovary, Lip: Lip; Gy: gynostemium) of *D. chrysotoxum* at three flower development stages (S1: unpigmented bud stage; S2: pigmented bud stage; S3: initial bloom stage). The FPKM values of *PEBP*s are in Table S3.

The transcripts are enriched based on their functional gene expression profiles. According to the FPKM value, the expression levels of 11 kinds of *DchPEBP*s in different parts and development stages (Figure 5) show that *DchPEBP7* is highly expressed at the S2 stage compared to the other two stages, *DchPEBP8* is highly expressed in the sepal part of the S1 stage, and *DchPEBP9* is highly expressed in the ovary part of the S1 stage. *DchPEBP3* is highly expressed in the S3 period, and *DchPEBP11* is highly expressed in the S1, S2, and S3 periods. *DchPEBP7* belongs to the TFL subfamily, and both *DchPEBP8* and *DchPEBP9* belong to the FTL subfamily and exhibit similar expression patterns during flower development. There were differences in *DchPEBP* gene expression, which suggested that the *DchPEBP* gene might have different functions in terms of regulating the flowering of *D. chrysotoxum*.

### 2.7. RT-qPCR Analysis of PEBP Genes in D. chrysotoxum

**Figure 6:**
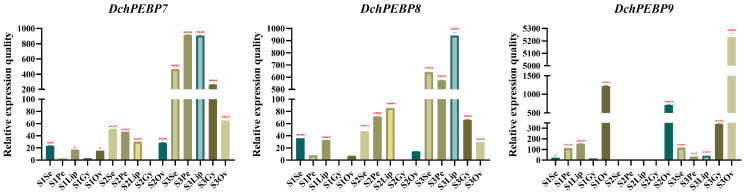
Real-time reverse transcription quantitative PCR (RT-qPCR) verifies the effect of *PEBP*s on flower organ development. Y-axis represents relative expression values (2^−ΔΔCT^). Bars represent the mean values of three technical replicates ± SE. The red asterisk indicates the *p* value in the significance test (* *p* < 0.05, *** *p* < 0.001, **** *p* < 0.0001).

In order to study the expression patterns of the PEBP gene family in five parts of the *D. chrysotoxum* during flower development, candidate genes with large expression differences were selected (Figure 6). Two genes from the FT subfamilies *DchPEBP8* and *DchPEBP9* and TFL subfamily *DchPEBP7*, which play an important role in flower development, were analyzed quantitatively with real-time PCR (RT-qPCR). The expression of *DchPEBP8* and *DchPEBP9* genes was up-regulated in petals, ovary, and other parts during S1 and S2, which may be involved in promoting the flowering of *D. chrysotoxum*. Meanwhile, the expression of *DchPEBP7* was up-regulated in various parts of the S3 stage, which may inhibit flowering. The expression of *DchPEBP9* was up-regulated in the ovary of S1, S2, and S3, which may be involved in promoting *D. chrysotoxum* morphogenesis. 

## 3. Discussion

Its members consist of genes that play vital roles in flowering and plant structure [13,28]. The process of gene family formation can be impacted by tandem and fragment duplications [17,29]. Twenty-two chromosomes were found in the *D. chrysotoxum* and *D. nobile* and were unevenly distributed across the chromosomes. In these studies, 22 *PEBPs* were found in the *D. chrysotoxum* and *D. nobile* genomes. These PEBP genes were not evenly distributed on these chromosomes, which suggests potential functional similarities and differences between them. By collinear analysis, three collinear pairs of *PEBP*s were identified in two species of *Dendrobium* chinensis, respectively, indicating that the PEBP gene had fragment replication. *D. chrysotoxum* and *D. nobile* have the same number of genes, the same tandem repeats, similar branches, and similar collinear positions, which indicates that the PEBP protein family has a high degree of similarity in terms of evolutionary relationship and sequence conservation.

Phylogenetic trees were constructed for 45 PEBP domain proteins in *D. chrysotoxum*, *D. nobile*, *O. sativa*, and *A. thaliana*, revealing subpopulations of genes with similar functions. The number of genes in the PEBP gene family was the same in *D. chrysotoxum* and *D. nobile*, and the two species of *Dendrobium* were more closely related to *O. sativa*. We also found five conserved motifs of *D. chrysotoxum* and *D. nobile*, and the PEBP protein in the same group had similar motifs (Figure 2). These findings suggest that the PEBP gene predates the divergence of monocots and dicots. The MFT and TFL subfamilies all have the same motif1,2,3,4,5. In addition to motif deletion in *DchPEBP4*, *DnoPEBP5,* and *DchPEBP6*, other genes in the FT subfamily contain motif1,2,3,4,5. This may have been caused by evolution. Most of the PEBP genes identified in this study contained four exons and three introns, and all PEBP genes showed a conserved gene structure, supporting close evolutionary relationships. These characteristics may imply functional similarities and differences between PEBP.

*Cis*-acting regulatory elements in the promoter region regulate gene expression and function, and are common to most *PEBP*s. These stress, hormone, and photonics-acting elements are common to most *PEBP*s, and the expression of most *PEBP*s is induced by ABA, JA, and light. There are typical light response elements AE-box, Box 4, G-box, and TCT-motif, which are analyzed similarly to assessing *PbFT* expression in pears [30]. The expression of the *PbFT* gene may be regulated by light and is most abundant in photo-responsive components. Thus, we speculated that the expression of the FT gene in *D. chrysotoxum* might also be regulated by light. Therefore, these results suggest that *PEBP*s may be involved in flowering time control, photoresponse, and hormonal response.

On the basis of transcriptome data, and to further analyze the role of the PEBP gene in *D. chrysotoxum*, we selected three PEBP expressed in *D. chrysotoxum* for RT-qPCR assay. The expression trend of these genes was similar to that of the transcriptome. The expression of these genes in all the flower organs of *D. chrysotoxum* S3 was significantly up-regulated. The expression of the *PhFT-1* gene in *Phalaenopsis* ‘V31’ is similar to that found in buds [31]. Furthermore, overexpression of *PhFT-1* in transcripts of *Arabidopsis* causes early flowering. These results suggest that the *PhFT-1* transcript may play a role in the flowering process of *Phalaenopsis* orchids, which supports our hypothesis. Thus, these two genes may play a role in the development of *D. chrysotoxum* (Figure 6). RT-qPCR results showed that the expression of *DchPEBP8* and *DchPEBP9* genes was up-regulated in the petals, ovary, and other parts during S1 and S2, which may be involved in promoting the flowering of *D. chrysotoxum*. Meanwhile, the expression of *DchPEBP7* was up-regulated during various parts of the S3 stage, which may inhibit flowering. In apple (*Malus domestica* Borkh), *MdFT1* and *MdFT2* are specifically expressed in the terminal bud and flower organs, respectively, and *MdFT1* and *MdFT2* may play an upstream role in regulating apple flowering [32]. The PEBP gene family has been studied, and the expression of the *OnFT* gene was found to be highest in axillary bud and bud organs, followed by that in the stem, with the lowest level in the root [33]. The gene known as FT is found in cauliflower’s stem, bud, and silique tissues, while the TFL1 gene is more commonly expressed in the reproductive organs and has the highest level of transcription in the silique and curd tissues, respectively. It is possible that the TFL1 gene’s high expression in the curd may have inhibited the expression of the flowering induction genes such as FT and TSF [34], which is consistent with our conclusion that high expression of *DchPEBP7* may inhibit flowering. The high expression of *DoFT* in the gynandrium of *D.* ‘Chao Praya Smile’ indicates that *DoFT* is a necessary gene for orchid reproductive development [18]. This is similar to our hypothesis that the expression of *DchPEBP9* was up-regulated in the ovary of S1, S2, and S3, which may be involved in promoting *D. chrysotoxum* morphogenesis. Studies have shown that FT genes induce flowering in plants. The principal function of TFL1 is to inhibit flower formation and maintain the infinite growth of the inflorescence meristem [35].

## 4. Materials and Methods

### 4.1. Plant Materials

The plant material selected for this study was obtained from wild types grown under natural conditions in the greenhouse of the Forest Orchid Garden of Fujian Agriculture and Forestry University. The plants were grown at an altitude of 10 m, and the temperature ranged between 25 °C and 30 °C. Samples of the flower parts (sepal, Se; petal, Pe; lip, Lip; and gynostemium, Gy) for each of the three flower development stages (S1: unpigmented bud stage; S2: pigmented bud stage; S3: initial bloom stage) of *D. chrysotoxum* and *D. nobile* (Sequences in Figure S1) were sampled with liquid nitrogen and stored in a refrigerator at −80 °C.

### 4.2. Identification and Physicochemical Properties of the PEBPs

The genome files for the two *Dendrobium* species were compared using TBtools v2.008 and Blast Compare Two Seqs, with 6 *AthPEBP*s (PEBP genes of *A. thaliana*) as probes (E-value, 1 × 10^−5^) [36,37], and the possible sequences obtained were blasted again (https://blast.ncbi.nlm.nih.gov/Blast.cgi?PROGRAM=blastp&PAGE_TYPE=BlastSearch&LINK_LOC=blasthome, accessed on 11 September 2023) in NCBI. To identify the conserved domains of PEBP, we downloaded the relevant data from an online database [3] (http://pfam.xfam.org/, accessed on 11 September 2023) and conducted an HMMER search using default parameters. The genes obtained from this search were then analyzed using NCBI Batch CDD (https://www.ncbi.nlm.nih.gov/Structure/bwrpsb/bwrpsb.cgi, accessed on 11 September 2023). We aimed to identify and retain only the genes with complete PEBP domains, based on the results of the Blast and HMMER searches [38]. The physical and chemical properties of the proteins were analyzed using the online analysis software ExPASy (https://www.expasy.org/, accessed on 11 September 2023). This included examining important metrics, such as protein length, isoelectric point (pI), molecular weight (MW), hydrophilic large average (GRAVY), instability index (II), and fat index (AI) [39].

### 4.3. Phylogenetic Analysis

This study compared 11 PEBP proteins of *D. chrysotoxum* (*DchPEBP*), 11 PEBP proteins of *D. nobile* (*DnoPEBP*), 6 PEBP proteins of *A. thaliana* (*AthPEBP*), and 19 PEBP proteins of *O. sativa* (*OsPEBP*) introduced into the ClustalW program in MEGA 7.0 [10,40]. with Gap Opening and Gap Extend values of 15 and 6.66, respectively, and the DNA Weight Matrix selection was set to IUB. The Phylogeny test was performed using 500 replications of the bootstrap method [41]. The resulting phylogenetic tree was then improved and beautified using the online software Evloview 3.0 (https://www.evolgenius.info/evolview-v2/#mytrees/1/2, accessed on 16 September 2023) [42].

### 4.4. Protein Conservative Domain and Gene Structure Analysis

The conserved motifs of PEBP proteins from *D. chrysotoxum* and *D. nobile* were analyzed using the MEME online software (https://meme-suite.org/meme/tools/meme/, accessed on 11 September 2023)with the prediction number set to ten [43]. Additionally, TBtools v2.008 was utilized to integrate gene structures, conserved protein motifs, and phylogenetic trees for general comparative mapping purposes. The software proved to be a valuable tool in this process.

### 4.5. Collinearity and Location Analysis on Chromosome

We used TBtools v2.008 software to extract the location information of the PEBP genes from the genome and gene annotation files of *D. chrysotoxum* and *D. nobile*. This helped us to construct a physical map of the *PEBP*s from both *Dendrobium* species detailing their respective chromosomes. To analyze collinearity, we compared the genome data of both species using the One Step MCScanx program in TBtools v2.008. Finally, we visualized the duplication patterns in both *Dendrobium* species using the One Step MCScanx program and Advance Circos.

### 4.6. Promoter Element Analysis of PEBPs

We utilized TBtools to extract a 2000 bp gene sequence located upstream of the promoter codon from the genomes of two *Dendrobium* species. This was performed in order to identify potential *cis*-elements present in the promoter [14]. To analyze *cis*-regulatory elements in the promoter region of *PEBP*s in the two species of *Dendrobium*, we employed PlantCARE online software (https://bioinformatics.psb.ugent.be/webtools/plantcare/html/, accessed on 12 September 2023). Finally, we processed and sorted the data using Excel 2020 software before visualizing it with TBtools v2.008.

### 4.7. Expression Pattern and RT-qPCR Analysis

To investigate the expression pattern of the *PEBP*s during floral development in *D. chrysotoxum*, we used RNA-Seq by Expectation Maximization (RSEM) [44] for transcription quantification. We calculated the fragments per kilobase per million mapped reads (FPKM) for each gene and established the transcriptome database of flower parts at different stages (three replicates were set for each sample). Finally, we created a heat map using TBtools based on the FPKM values.

The expression pattern of the *PEBP*s was analyzed using RT-qPCR. Total RNA was extracted from the flower parts of *D. chrysotoxum* during three periods of flowering using a FastPure Plant Total RNA Isolation Kit (for polysaccharide- and polyphenol-rich tissues) from Vazyme Biotech Co., Ltd., Nanjing, China. First-strand DNA was synthesized with TransScript^®^ All-in-One First-Strand cDNA Synthesis SuperMix for quantitative PCR (TransGen Biotech, Beijing, China). Primers for candidate and internal reference genes for RT-qPCR were designed using Primer Premier 5.0 software. Primer specificity was confirmed using a primer blast on the NCBI website. RT-qPCR assays were performed using Hieff^®^ qPCR SYBR Green Master Mix (Low Rox Plus) from Yeasen Biotechnology (Shanghai) Co., Ltd., Shanghai, China. The Maker75111 served as the reference gene (Table S4). Finally, the relative expression of the target genes was calculated using the 2^−ΔΔCT^ method (using Gy2 as a reference). The expression data were the mean of the three biological replicates [45].

## 5. Conclusions

We have identified 11 DchPEBPs and 11 DnoPEBPs in two species of Dendrobium, which have been classified into three subfamilies based on their phylogenetic relationships. The PEBP genes in these two species have been analyzed for their genome-wide identification, phylogeny, functional classification, gene structure, motif composition, and chromosomal localization. Our research highlighted the varying transcription patterns of DchPEBPs in different parts of the flower during its development. We found that DchPEBP8 and DchPEBP9 may regulate downstream genes to promote flowering, while DchPEBP7 may inhibit flowering. Our findings provide crucial insights for further studies on the regulatory mechanisms and functional roles of PEBP genes in different parts of Dendrobium flower development.

## Data Availability

The sequence data used in the study can be found in Table S1. The *AthPEBP* sequences were downloaded from PlantTFDB (http://planttfdb.gao-lab.org/, accessed on 11 August 2023) and *OsPEBP* sequences were downloaded from NCBI (https://www.ncbi.nlm.nih.gov/, accessed on 11 August 2023).

## Supplementary Materials

The following supporting information can be downloaded at: https://www.mdpi.com/article/10.3390/ijms242417463/s1.
