# Supplementary material for: Genome-Wide Identification of PEBP Gene Family in Two Dendrobium Species and Expression Patterns in Dendrobium chrysotoxum
Source: Int J Mol Sci. 2023 Dec 14;24(24):17463. doi: 10.3390/ijms242417463 (PMC10743876; doi:10.3390/ijms242417463)
Supplement: Supplementary file 1 [file ijms-24-17463-s001.zip › Figure S1.pdf]

Supplementary Figure

(A)

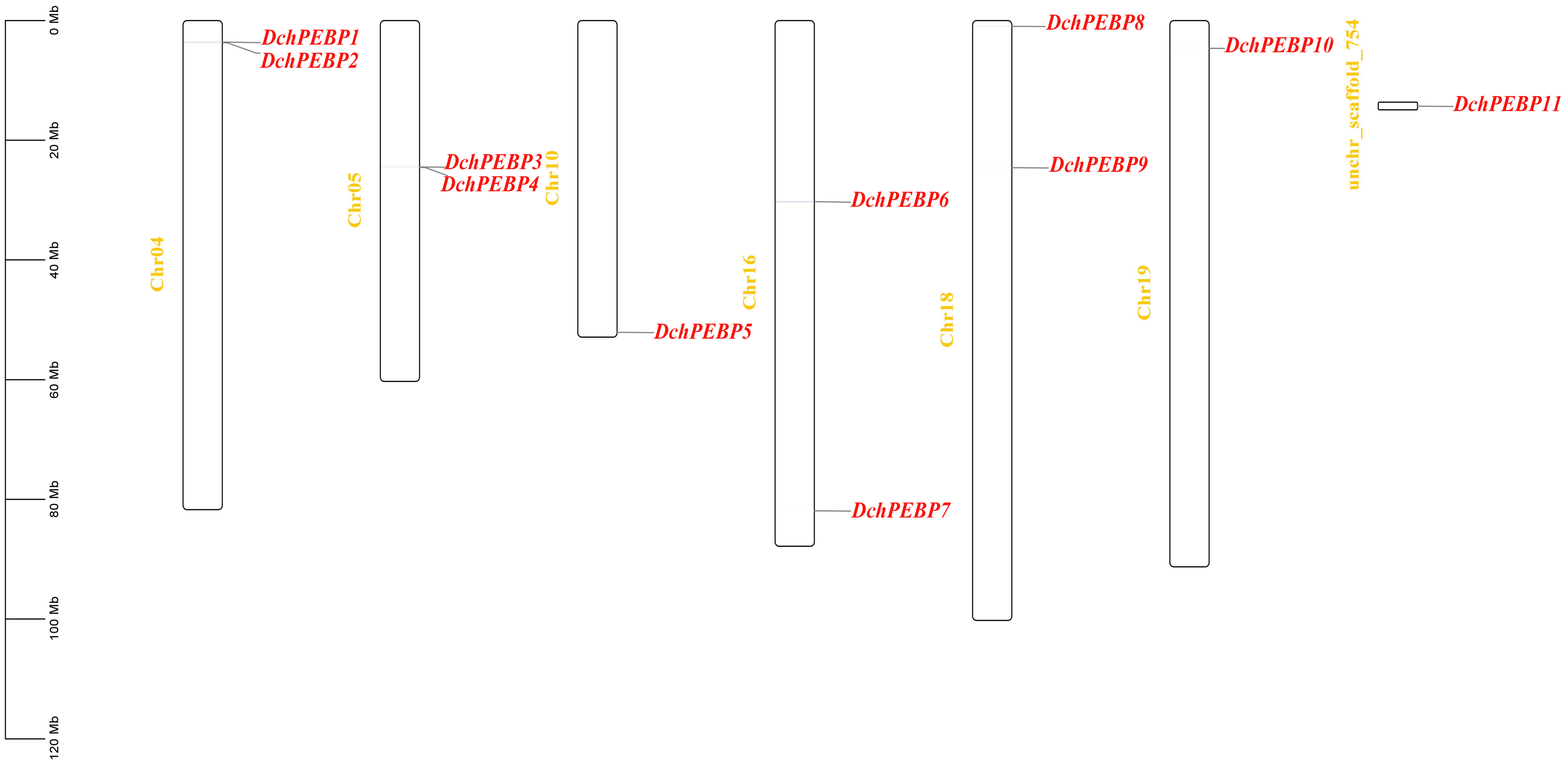

(B)

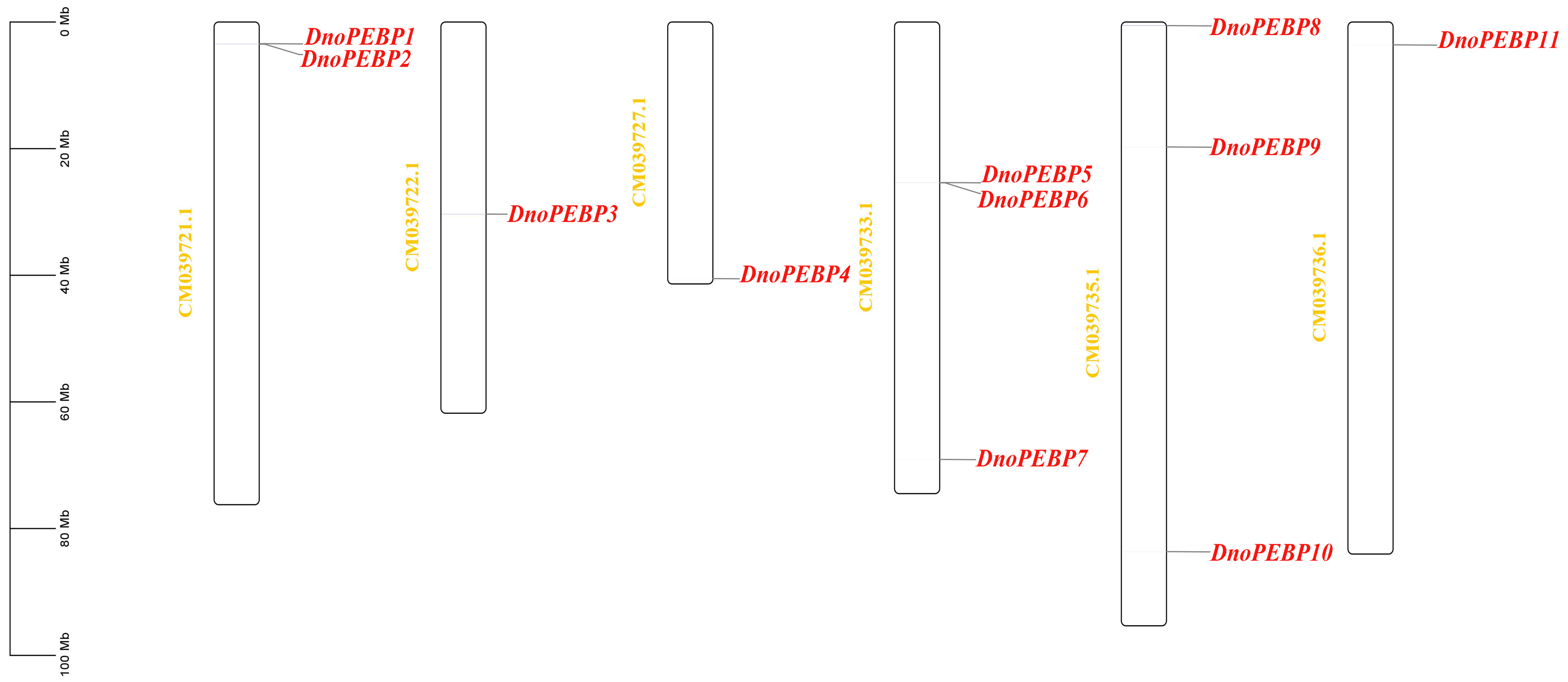

Figure S1. ***PEBP*s distribution on chromosomes of *D. chrysotoxum*(A), *D.nobile*(B).**
